# Optimizing treatment management of trastuzumab deruxtecan in clinical practice of breast cancer

**DOI:** 10.1016/j.esmoop.2022.100553

**Published:** 2022-08-11

**Authors:** H.S. Rugo, G. Bianchini, J. Cortes, J.-W. Henning, M. Untch

**Affiliations:** 1 University of California San Francisco Helen Diller Family Comprehensive Cancer Center, San Francisco, USA; 2 IRCCS Ospedale San Raffaele, Milan, Italy; 3 Università Vita-Salute San Raffaele, Milan, Italy; 4 Oncology Department, International Breast Cancer Center (IBCC), Quiron Group, Barcelona, Spain; 5 Medica Scientia Innovation Research (MedSIR), Barcelona, Spain; 6 Medica Scientia Innovation Research (MedSIR), Ridgewood, USA; 7 Faculty of Biomedical and Health Sciences, Department of Medicine, Universidad Europea de Madrid, Madrid, Spain; 8 Tom Baker Cancer Centre, University of Calgary, Calgary, Canada; 9 Helios Klinikum Berlin-Buch, Berlin, Germany

**Keywords:** trastuzumab deruxtecan, adverse event, breast cancer, nausea, vomiting, interstitial lung disease

## Abstract

**Introduction:**

The antibody-drug conjugate trastuzumab deruxtecan (T-DXd) targets human epidermal growth factor receptor 2 (HER2) and has been evaluated in patients with HER2-positive unresectable/metastatic breast cancer in the phase II DESTINY-Breast01 trial (NCT03248492; DS8201-A-U201) and the randomized phase III DESTINY-Breast03 trial (NCT03529110; DS8201-A-U302). Approximately 20 additional studies are ongoing in breast cancer, including HER2-low breast cancer, and other solid tumor types within the DESTINY trial program. T-DXd has demonstrated a generally manageable safety profile, with low-grade hematologic and gastrointestinal adverse events (AEs) among the most common; interstitial lung disease (ILD)/pneumonitis has been observed in patients receiving T-DXd and can be severe. This review discusses the management of common AEs and AEs of special interest in patients with HER2-positive unresectable/metastatic breast cancer, including nausea and vomiting, neutropenia, infusion-related reactions, alopecia, fatigue, ILD/pneumonitis, and left ventricular dysfunction.

**Methods:**

Expert opinions, institutional protocols, and strategies to help optimize AE management and maximize the potential benefits of T-DXd in patients with breast cancer from five oncologists treating patients with T-DXd in North America and Europe are discussed.

**Results:**

Prophylaxis for nausea and vomiting and proactive management of ILD/pneumonitis are especially important in treating patients with T-DXd. Management strategies for other T-DXd-related AEs of interest (e.g. neutropenia, infusion-related reactions, alopecia, fatigue, and left ventricular dysfunction) are also discussed.

**Conclusions:**

This review provides context for understanding the usage, monitoring, and management practices of other health care providers and institutions with experience using T-DXd to help with safe and effective management of T-DXd-related AEs, particularly since the duration of T-DXd treatment may be quite long. Proper management of T-DXd-related AEs will allow optimal exposure and benefit from T-DXd and will help avoid premature discontinuation or improper dose reductions.

## Highlights

• Adverse events (AEs) related to trastuzumab deruxtecan (T-DXd) are frequent but most commonly low grade and manageable*.*
• Nausea and vomiting are among the most common treatment-emergent AEs*.*
• Interstitial lung disease/pneumonitis is an important AE; proactive monitoring, diagnosis, and management are key*.*
• Optimizing management of AEs with prophylaxis and effective therapies allows for maximizing the benefit from T-DXd therapy*.*

## Introduction

Trastuzumab deruxtecan (T-DXd) is a human epidermal growth factor receptor 2 (HER2)-targeted antibody-drug conjugate composed of an antibody, a tetrapeptide-based cleavable linker, and a topoisomerase I inhibitor payload (DXd).^1^^,^^2^ The linker is selectively cleaved by cathepsins up-regulated in cancer cells,^2^, ^3^, ^4^, ^5^ releasing the payload. The payload, which is membrane permeable, allows for cytotoxic effects on nearby cancer cells by a so-called bystander effect, with a short half-life to minimize systemic exposure.^2^^,^^4^

T-DXd was evaluated in patients with HER2-positive unresectable or metastatic breast cancer who had previously been treated with trastuzumab emtansine (T-DM1) in the registrational, phase II, open-label DESTINY-Breast01 trial (NCT03248492; DS8201-A-U201).^6^, ^7^, ^8^ T-DXd demonstrated a confirmed objective response rate (ORR) of 62.0% (114 of 184 patients treated at 5.4 mg/kg every 3 weeks) and durable benefit {median duration of response, 18.2 months [95% confidence interval (CI) 15.0 months to not evaluable]; median progression-free survival (PFS), 19.4 months (95% CI 14.1 to 25.0 months)} in a heavily pretreated patient population (median of six prior treatments; all patients had previous disease progression on T-DM1); only 1.6% (3 of 184 patients) had disease progression.^7^^,^^8^ Data from DESTINY-Breast01 supported global approvals of T-DXd for use in adult patients with unresectable or metastatic HER2-positive breast cancer that progressed on two or more prior therapies.^9^, ^10^, ^11^, ^12^, ^13^, ^14^, ^15^

In May 2022, the breast cancer indication for T-DXd was expanded in the USA to include use in adult patients with unresectable or metastatic HER2-positive breast cancer who received treatment with a prior anti-HER2-based regimen in the metastatic setting or in the neoadjuvant or adjuvant setting and who developed disease recurrence during or within 6 months of completing therapy.^11^ This approval was based on data from the randomized phase III DESTINY-Breast03 trial of T-DXd versus T-DM1 (NCT03529110; DS8201-A-U302) in patients with HER2-positive unresectable/metastatic breast cancer who had previously received trastuzumab plus a taxane.^16^ T-DXd monotherapy demonstrated efficacy versus T-DM1, with a clinically meaningful and significant improvement in PFS: a 12-month PFS rate of 75.8% (95% CI 69.8% to 80.7%) with T-DXd versus 34.1% (95% CI 27.7% to 40.5%) with T-DM1 [hazard ratio (HR), 0.28; *P* < 0.001].^16^ The ORR was 79.7% (95% CI 74.3% to 84.4%) with T-DXd versus 34.2% (95% CI 28.5% to 40.3%) with T-DM1, with 1.1% versus 17.5% of patients, respectively, having progressive disease.^16^ An encouraging trend in overall survival (OS) was also observed, with a 12-month OS rate of 94.1% (95% CI 90.3% to 96.4%) with T-DXd versus 85.9% (95% CI 80.9% to 89.7%) with T-DM1 [HR, 0.55 (95% CI 0.36 to 0.86); *P* = 0.007, which does not cross the prespecified boundary of *P* < 0.000265 for this analysis].^16^

The benefit of T-DXd treatment has also been demonstrated via patient-reported outcomes from the DESTINY-Breast03 trial.^17^ Overall health status and quality of life were maintained with T-DXd compared with T-DM-1 as measured by the European Organisation for Research and Treatment of Cancer Quality of Life Core 30 Questionnaire (EORTC QLQ-C30); mean change from baseline was 1.98 with T-DXd and 4.07 with T-DM1.^17^ Treatment with T-DXd also delayed deterioration across all EORTC QLQ-C30 subscales (i.e. global health status/quality of life, pain symptoms, and physical, emotional, and social functioning), with all HRs numerically favoring T-DXd over T-DM1 (HR range, 0.69-0.90).^17^

T-DXd (5.4 mg/kg or 6.4 mg/kg every 3 weeks) has also been previously evaluated in 115 patients with HER2-positive unresectable or metastatic breast cancer from the USA or Japan [DS8201-A-J101 (NCT02564900)].^18^ T-DXd had a confirmed ORR of 59.5% (66 of 111 assessable patients; 95% CI 49.7% to 68.7%) with a median duration of response of 20.7 months (95% CI, not estimable); median PFS was 22.1 months (95% CI, not estimable) in this patient population, which had received a median of 7 (range, 5-11) prior lines of therapy including T-DM1.^18^

Additionally, data from the randomized, phase II DESTINY-Gastric01 study (NCT03329690; DS8201-A-J202) led to approvals in multiple countries of T-DXd (6.4 mg/kg every 3 weeks) for locally advanced or metastatic HER2-positive gastric cancer.^10^^,^^11^^,^^14^^,^^15^^,^^19^ Clinical investigation of T-DXd is also ongoing in several other patient populations, including patients with HER2-low breast cancer, HER2-positive or mutated metastatic non-small-cell lung cancer, or HER2-expressing advanced colorectal cancer, and those with early disease, among others.^20^, ^21^, ^22^, ^23^, ^24^, ^25^, ^26^

Recently, results from the randomized, phase III, open-label, DESTINY-Breast04 trial (NCT03734029; DS8201-A-U303) evaluating T-DXd in patients with HER2-low [defined as an immunohistochemistry (IHC) score of 1+ or IHC 2+ with a negative *in situ* hybridization score] unresectable or metastatic breast cancer previously treated with one or two prior lines of chemotherapy were reported.^21^ T-DXd (5.4 mg/kg every 3 weeks) demonstrated a significant improvement in PFS and OS versus physician’s choice of chemotherapy in this HER2-low metastatic breast cancer population.^21^ Demonstrating efficacy of T-DXd outside the classically defined HER2-overexpressing/HER2-amplified tumors and across a broad spectrum of tumor types (e.g. breast, gastric, lung, colorectal) would represent a paradigm shift for HER2-targeted therapies.

Across the DESTINY clinical program, T-DXd has demonstrated a generally manageable and tolerable safety profile, with low-grade hematologic and gastrointestinal adverse events (AEs) being most common. In the DESTINY-Breast01 trial,^6^^,^^7^ 15.8% of patients (29 of 184) developed drug-related interstitial lung disease (ILD)/pneumonitis; most of these patients [79.3% (23 of 29)] experienced grade 1 or 2 cases. In the overall study population, 2.7% of patients (5 of 184) had a fatal event.^8^ In DESTINY-Breast03, drug-related AEs occurred in 98.1% of patients (252 of 257), with nausea [72.8% (187 of 257)], fatigue [44.7% (115 of 257)], vomiting [44.0% (113 of 257)], and neutropenia [42.8% (110 of 257)] being the most common AEs; ILD/pneumonitis occurred in 10.5% of patients (27 of 257; all grade ≤3).^16^ In J101, the most common AEs were nausea [79% (81 of 115)], decreased appetite [56% (64 of 115)], and vomiting [52% (60 of 115)]; ILD/pneumonitis occurred in 9.6% (11 of 115), with all being grade ≤3.^18^ In DESTINY-Gastric01, all patients had one or more AE, with nausea [63% (79 of 125)], neutropenia [63% (79 of 125)], decreased appetite [60% (75 of 125)], and anemia [58% (72 of 125)] being the most common; ILD/pneumonitis occurred in 10% of patients (12 of 125; grade 1 or 2, *n* = 7; no fatal events).^19^ ILD/pneumonitis is considered one of the AEs of special interest with T-DXd, and it can be life-threatening^6^^,^^21^^,^^27^; it appears in a warning on approved labels for T-DXd.^9^, ^10^, ^11^, ^12^, ^13^, ^14^, ^15^ Other warnings and precautions associated with T-DXd include left ventricular dysfunction, neutropenia, embryofetal toxicity, and hepatic impairment.^9^, ^10^, ^11^, ^12^, ^13^, ^14^, ^15^

The objective of this review is to provide expert perspectives and insights based on our experience regarding AE prophylaxis, monitoring, and management of patients with breast cancer treated with T-DXd. Proper management is needed to optimize the effectiveness of T-DXd treatment and reduce the number of serious AEs.

## What to expect with t-dxd infusions

**Figure 1:**
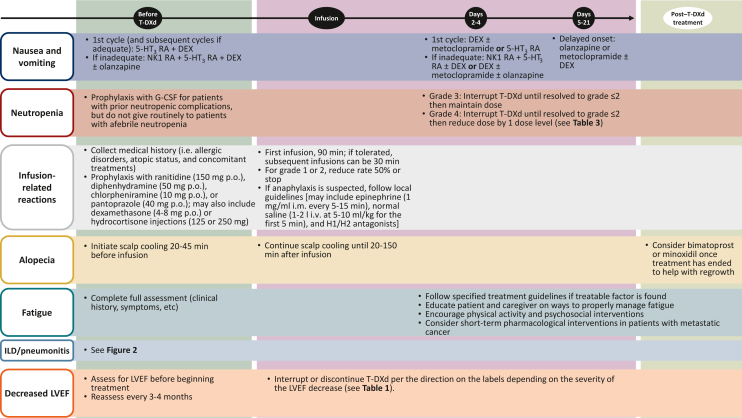
**Overview of management of T-DXd-related adverse events.** 5-HT_3_, serotonin type 3; DEX, dexamethasone; G-CSF, granulocyte colony-stimulating factor; ILD, interstitial lung disease; i.m., intramuscular; i.v., intravenous; LVEF, left ventricular ejection fraction; NK1, neurokinin-1; p.o., by mouth; RA, receptor antagonist; T-DXd, trastuzumab deruxtecan.

T-DXd is administered intravenously (i.v.) in a hospital or a clinic. Education about expected AEs before starting therapy is critical.^11^ Prophylaxis for nausea and vomiting should be administered using a regimen that has been selected according to the patient’s risk for emesis, with a standard initial regimen, including dexamethasone and a serotonin type 3 (5-HT_3_) receptor antagonist, which can be tapered or escalated based on individual tolerance (Figure 1). The first infusion of T-DXd should be given over 90 min, with subsequent infusions given over 30 min if well tolerated. Infusion-related reactions have occurred with T-DXd in 1%-3% of patients and can be managed by slowing the infusion rate and increasing premedications as clinically indicated.^6^^,^^9^^,^^11^^,^^13^, ^14^, ^15^ The infusion clinic should be equipped to properly handle acute management of such reactions, as well as prophylaxis for future infusions. After the infusion, patients should continue with antiemetics as needed as described in the subsequent sections.

## Management of common t-dxd-related aes

### Nausea and vomiting

Gastrointestinal AEs are among the most common side-effects observed with T-DXd treatment, with nausea being the most frequently reported (Table 1).^6^^,^^16^^,^^19^ In DESTINY-Breast03, any-grade and grade ≥3 nausea occurred in 72.8% (187 of 257) and 6.6% (17 of 257) of patients, respectively.^16^ Vomiting is also commonly reported, with 44.0% (113 of 257) and 1.6% (4 of 257) of patients experiencing any-grade and grade ≥3 vomiting, respectively.^16^ Based on these reported incidence rates, T-DXd would be classified as having a moderate emetic risk according to the National Comprehensive Cancer Network Clinical Practice in Oncology Guidelines on antiemesis.^28^^,^^29^ The category encompassing ‘moderately emetogenic’ spans a broad range; thus, antiemetic prophylaxis, though standardized, may frequently require individual optimization.^29^^,^^35^

**Table 1:** Summary of AEs and their management

Event	Frequency in clinical trials of breast cancer	Management
Nausea and vomiting	• DESTINY-Breast03, 72.8% (187/257) and 44.0% (113/257) [grade ≥3, 6.6% (17/257) and 1.6% (4/257)], respectively^16^ • DESTINY-Breast01, 77.7% (143/184) and 45.7% (84/184) [grade ≥3, 7.6% (14/184) and 4.3% (8/184)], respectively^6^	• Pretreatment with a 5-HT_3_ receptor antagonist and dexamethasone with or without a neurokinin-1 receptor antagonist^28^, ^29^, ^30^ • Delayed nausea prophylaxis: give dexamethasone on days 2-3 after infusion of T-DXd^28^, ^29^, ^30^ • Grade 3: delay dose until resolved to grade ≤1^31^ • If resolved in ≤7 days, maintain dose • If resolved in >7 days, reduce dose 1 level
Neutropenia	• DESTINY-Breast03, 42.8% (110/257) [grade ≥3, 19.1% (49/257)]^16^ • DESTINY-Breast01, 34.8% (64/184) [grade ≥3, 20.7% (38/184)]^6^	• Grade 3: hold T-DXd until resolved to grade ≤2, then maintain dose^9^, ^10^, ^11^, ^12^, ^13^, ^14^, ^15^ • Grade 4: hold T-DXd until resolved to grade ≤2, then reduce dose 1 level (Table 3)^9^, ^10^, ^11^, ^12^, ^13^, ^14^, ^15^
Febrile neutropenia	• DESTINY-Breast01, 1.6% (3/184)^6^	• Hold T-DXd until resolved, then reduce dose 1 level (Table 3)^9^, ^10^, ^11^, ^12^, ^13^, ^14^, ^15^
Thrombocytopenia	• DESTINY-Breast03, 24.9% (64/257) [grade ≥3, 7.0% (18/257)]^16^ • DESTINY-Breast01, 21.2% (39/184) [grade ≥3, 4.3% (8/184)]^6^	• Grade 3: hold T-DXd until resolved to grade ≤1, then reduce or maintain dose^a^^,^^10^^,^^11^^,^^14^ • Grade 4: hold T-DXd until resolved to grade ≤1, then reduce dose 1 level (Table 3)^10^^,^^11^^,^^14^
Infusion-related reactions	• DESTINY-Breast01, 2.2% (4/184) (grade ≥3, 0%)^6^	• First infusion should be administered over 90 min; subsequent infusions may be given over 30 min if prior infusions were well tolerated^9^, ^10^, ^11^, ^12^, ^13^, ^14^, ^15^ • The infusion rate may be reduced to 50% or temporarily stopped if a patient experiences an infusion-related reaction (grade 1 or grade 2, respectively); the infusion should be permanently stopped if the reaction is severe (grade 3 or 4)^9^, ^10^, ^11^, ^12^, ^13^, ^14^, ^15^^,^^32^
Alopecia	• DESTINY-Breast03, 36.2% (93/257)^16^ • DESTINY-Breast01, 48.4% (89/184)^6^	• Suggest scalp hypothermia treatment to prevent alopecia in patients with breast cancer
Fatigue	• DESTINY-Breast03, 44.7% (115/257) [grade ≥3, 5.1% (13/257)]^16^ • DESTINY-Breast01, 49.5% (91/184) [grade ≥3, 6.0% (11/184)]^6^	• Investigate and rule out treatable causes for fatigue • If present, treat accordingly • Patients should be educated regarding expectations for cancer-related fatigue^33^^,^^34^ • Dose reduction of T-DXd could be considered if T-DXd-related fatigue occurs
ILD/pneumonitis	• DESTINY-Breast03, 10.5% (27/257) [grade ≥3, 0.8% (2/257)]^16^ • DESTINY-Breast01, 15.8% (29/184) [grade ≥3, 3.2% (6/184)]^8^	• Asymptomatic/grade 1: hold T-DXd until resolved to grade 0, then^b^^,^^9^^,^^11^, ^12^, ^13^, ^14^, ^15^: • If resolved in ≤28 days, maintain dose • If resolved in >28 days, reduce dose 1 level (Table 3) • Consider corticosteroid treatment at onset • Symptomatic/grade ≥2: permanently discontinue T-DXd and begin corticosteroid treatment^9^, ^10^, ^11^, ^12^, ^13^, ^14^, ^15^ • Follow monitoring and management guidelines as previously described^20^
LVEF decrease	• DESTINY-Breast03, 2.7% (7/257) (grade ≥3, 0%)^16^ • DESTINY-Breast01, 2.2% (4/184) [grade ≥3, 0.5% (1/184)]^7^	• LVEF >45% and absolute decrease from baseline of 10%-20%: continue treatment^9^, ^10^, ^11^, ^12^, ^13^, ^14^, ^15^ • LVEF 40%-45% and absolute decrease from baseline <10%: continue treatment^c^ and repeat LVEF assessment within 3 weeks^9^^,^^11^, ^12^, ^13^, ^14^, ^15^ • LVEF 40%-45% and absolute decrease from baseline 10%-20%: hold treatment; repeat LVEF assessment within 3 weeks. If LVEF has not recovered to within 10% of baseline, permanently discontinue^9^, ^10^, ^11^, ^12^, ^13^, ^14^, ^15^ • LVEF <40% or absolute decrease from baseline >20%: hold treatment and repeat LVEF assessment within 3 weeks. If LVEF <40% or absolute decrease from baseline >20% is confirmed, permanently discontinue^9^, ^10^, ^11^, ^12^, ^13^, ^14^, ^15^ • Symptomatic CHF: permanently discontinue^9^, ^10^, ^11^, ^12^, ^13^, ^14^, ^15^

5-HT_3_, serotonin type 3; AE, adverse event; CHF, congestive heart failure; ILD, interstitial lung disease; LVEF, left ventricular ejection fraction; T-DXd, trastuzumab deruxtecan.

a In Japan, the prescribing information recommends to maintain the dose of T-DXd if resolved to grade ≤1 in ≤7 days and to reduce dose 1 level (Table 3) if resolved to grade ≤1 in >7 days.^10^

b In Japan, the prescribing information recommends permanently discontinuing T-DXd treatment in the event of any-grade ILD.^10^

c In Japan, the prescribing information recommends considering holding dose.^10^

Preventing and managing nausea and vomiting are important to improve patients’ experience with T-DXd. This issue is particularly significant because patients may remain on T-DXd treatment for an extended period of time^6^, ^7^, ^8^^,^^16^; optimal control of nausea and vomiting is imperative from the first treatment cycle. In addition, it is important to recognize that the pattern of nausea and vomiting may vary, with events occurring 1 week or more after each infusion. Therefore, attention needs to be paid to both prevention and treatment; optimal prophylaxis of acute and delayed nausea may also reduce the incidence of delayed nausea breakthrough in the middle of the cycle.

Several groups have published guidelines for antiemetic prophylaxis during cancer treatment, including the guidelines from the National Comprehensive Cancer Network, which are routinely updated.^28^, ^29^, ^30^^,^^36^^,^^37^ Also, an Italian expert panel recently published insights on the emetogenicity of antibody-drug conjugates, which focused on T-DXd and included information on antiemetic prophylaxis protocols used with this agent at a single center.^38^ Administering medication before T-DXd infusion is highly recommended.^38^ In patients without specific risk factors for emesis, the premedication regimen during the first cycle should initially consist of dexamethasone and a 5-HT_3_ receptor antagonist (Table 2).^28^^,^^30^^,^^37^^,^^38^ Based on individual tolerances and emesis risk, the regimen can be escalated or tapered. For high-risk patients (e.g. those with a history of emesis with other treatments) or frail patients, a combination of three drugs may be considered: a neurokinin-1 (NK1) receptor antagonist, dexamethasone, and a 5-HT_3_ receptor antagonist.^28^

**Table 2:** Example nausea and vomiting management protocol

Day	Medication protocols	Other considerations
Before infusion/day 1	• 1st cycle: dexamethasone (8-12 mg p.o. or i.v.) + 5-HT_3_ RA [e.g. palonosetron (0.25-0.5 mg i.v.), granisetron (10 mg s.c.), or ondansetron (8 mg i.v.)]	• For patients with anticipatory N/V, consider anxiolytic therapy [e.g. lorazepam (0.5-1.0 mg p.o.)] the night before infusion and 1-2 h before infusion begins^28^
	• Subsequent cycles: if optimal control, repeat above. If not (e.g. grade ≥1 for ≥3 days), dexamethasone (12 mg i.v.) + NK1 RA [aprepitant (125 mg p.o.) or netupitant (300 mg p.o.)] + 5-HT_3_ RA [e.g. palonosetron (0.25 mg i.v. or 0.5 mg p.o.) or granisetron (10 mg s.c.)]	• Behavioral therapy (e.g. relaxation exercises, hypnosis) and/or acupuncture/acupressure may also aid in anticipatory N/V prevention^38^^,^^39^
		• For subsequent infusions, estimate individual risk of emesis to determine whether past regimen was adequate or if escalation is necessary
After infusion/day 1	• Consider ondansetron (8 mg p.o. or i.v./i.m.) for 3 doses after infusion	• If N/V occur despite 3-drug regimen, offer olanzapine (2.5 mg p.o.; increase to 5-10 mg if needed) on days 1-4 or increase dexamethasone on days 2-4 on subsequent cycles
Days 2-4	• 1st cycle: dexamethasone (4 mg p.o. or 8 mg p.o. or i.v./i.m. daily) ± metoclopramide (10 mg p.o.) t.i.d. or 5-HT_3_ RA [e.g. granisetron (1-2 mg p.o. qd or 0.1 mg/kg i.v. qd)]	
	• Subsequent cycles: If adequate, repeat above. If not (e.g. grade ≥1 for ≥3 days), give aprepitant (80 mg p.o.) + 5-HT_3_ RA ± dexamethasone (8 mg p.o. or i.v.) or dexamethasone (8 mg p.o. or i.v./i.m. qd) ± metoclopramide (10 mg p.o. t.i.d.)	• For delayed nausea (after day 4), give olanzapine (5-10 mg p.o. at bedtime qd) or metoclopramide (10 mg p.o. t.i.d.) ± dexamethasone (4 mg p.o. qd) until resolution^38^

5-HT_3_, serotonin type 3; i.m., intramuscular; i.v., intravenous; N/V, nausea and/or vomiting; NK1, neurokinin-1; p.o., by mouth; q12h, every 12 h; qd, once daily; RA, receptor antagonist; s.c., subcutaneously; t.i.d., three times daily.

Regional differences exist in antiemetic protocols depending on drug access, institutional guidelines, and anecdotal experiences (e.g. metoclopramide is more common in Europe, and olanzapine is more common in the USA and Canada). In general, protocols include the following: on day 1 of the first cycle, administer dexamethasone (8-12 mg i.v.) along with a 5-HT_3_ receptor antagonist [e.g. palonosetron (0.25 mg i.v.), ondansetron (8 mg i.v.), or granisetron (10 mg subcutaneously)].^38^ On days 2-4, protocols suggest administering dexamethasone (4 mg by mouth or 8 mg by mouth/i.v. once a day) with or without metoclopramide (10 mg by mouth 3 times a day, if available) for 2-3 days until resolution; or give with a 5-HT_3_ receptor antagonist [e.g. granisetron (1-2 mg by mouth once a day or 0.1 mg/kg i.v. once a day)] for 2 days.^38^

For the second cycle, if management was adequate in the first cycle, repeat the previous regimen.^38^ If emesis was not extremely well controlled (e.g. grade ≥1 nausea for ≥3 days), it is highly recommended to immediately escalate to a three-drug regimen. For instance, we suggest on day 1 to give dexamethasone (12 mg i.v.) with an NK1 receptor antagonist [e.g. aprepitant (125 mg by mouth) with a 5-HT_3_ receptor antagonist or netupitant/palonosetron (300 mg/0.5 mg fixed combination product) by mouth].^38^ On days 2-4, treatment may vary but may include either aprepitant (80 mg by mouth) with or without dexamethasone (8 mg by mouth or i.v.) for 2 days or dexamethasone (4 or 8 mg by mouth once a day) with or without metoclopramide (10 mg by mouth three times a day) for 2-4 days until resolution based on institutional guidelines.^38^ Patients who experience nausea or vomiting despite the three-drug regimen should be offered the addition of olanzapine (2.5 mg by mouth; increased to 5 or 10 mg if needed) on days 1-4^38^ or an increase in dexamethasone on days 2-4. The recommended dose of olanzapine is 5-10 mg based on published guidelines^28^^,^^29^^,^^37^^,^^38^; however, we have found that 2.5 or 5 mg is sufficient for most patients.

Because T-DXd may result in a delayed presentation of nausea and vomiting, dexamethasone should also be offered prophylactically on days 2 and 3 after infusion and on day 4 if needed. In our experience, only some patients require medication beyond day 4. In these patients, we recommend administering olanzapine (5-10 mg by mouth once a day at bedtime) or metoclopramide (10 mg by mouth three times a day) with or without dexamethasone (4 mg by mouth once a day) until resolution. Each treatment option can present with added risk of toxicity. For example, olanzapine may cause somnolence, particularly with the 10-mg dose; metoclopramide may cause neurological side-effects, and therefore a maximum dose of 0.5 mg/kg/day is recommended.^28^^,^^29^^,^^35^^,^^37^

Protocols for T-DXd clinical trials were amended beginning in 2020 to recommend that patients receive prophylactic antiemetic agents such as 5-HT_3_ or NK1 receptor antagonists and/or steroids in accordance with the prescribing information and institutional guidelines.^20^ A recent case series looking at the management of nausea and vomiting in 10 patients receiving T-DXd treatment of HER2-positive breast cancer found that the rate of nausea and vomiting was 28.9% (13 of 45 doses) when patients were managed using a moderate-emetic-risk protocol, with all events being grade 1 or 2. When intervention was amended to the high-risk protocol (premedication using 130 mg aprepitant, 12 mg dexamethasone, and 16 mg ondansetron and take-home prescriptions of dexamethasone, ondansetron, and olanzapine), no instances of T-DXd-related nausea and vomiting were reported in the two patients treated (0 of 3 doses).^40^ If we took the approach of treating all patients receiving T-DXd using the high-emetic-risk protocol, it is expected that most would respond well, but a percentage of patients could possibly be overmedicated who also could have responded well to the moderate-emetic-risk protocol. Additionally, in our experience, even with treatment using the high-emetic-risk regimen, there may still be patients who do not respond well. In these cases where grade ≥3 events do not resolve within 7 days, it is advised to lower the dose of T-DXd.^31^ T-DXd-related nausea and vomiting, while common, can be effectively managed for the majority of patients when adequate antiemetic prophylaxis protocols are used.

### Neutropenia

In clinical trials, cytopenias were commonly seen in patients treated with T-DXd (Table 1).^1^^,^^6^^,^^16^^,^^19^^,^^20^^,^^27^^,^^41^ The most common cytopenia reported is neutropenia, but anemia, leukopenia, and thrombocytopenia also occur frequently.^6^^,^^16^^,^^19^ Neutropenia can be the result of either chemotherapy or the solid tumor malignancy,^42^ but it is also advisable to rule out concomitant medication interactions or comorbidities that can contribute to neutropenia. In DESTINY-Breast03, any-grade neutropenia occurred in 42.8% of patients (110 of 257), and grade ≥3 neutropenia occurred in 19.1% of patients (49 of 257).^16^ In DESTINY-Breast01, only 1.6% of patients (3 of 184) had febrile neutropenia.^6^

Neutropenia can be managed with dose reductions and holds until the event is resolved as indicated in the approved labels (Table 1).^9^, ^10^, ^11^, ^12^, ^13^, ^14^, ^15^ Duration and severity of neutropenia may also be reduced with the use of granulocyte colony-stimulating factor (G-CSF), which aids in white blood cell production within the bone marrow.^42^^,^^43^ Prophylaxis with G-CSF may be administered in patients with prior neutropenic complications, but it should not be routinely administered in patients with afebrile neutropenia.^44^ Also, because the risk of febrile neutropenia is ≤10% with T-DXd treatment, G-CSF prophylaxis is not indicated.^45^ While neutropenia may be quite common in patients receiving T-DXd, it is typically lower grade (grade 1 or 2) and does not require dose adjustment.

### Infusion-related reactions

Most infusion-related reactions to monoclonal antibodies, such as hypersensitivity, occur after the first infusion, with the likelihood of reaction declining with subsequent exposures.^46^ The mechanism of infusion reactions to monoclonal antibodies is not known.^46^ Known risk factors for anaphylactic reactions to anticancer drugs include age-related factors, concomitant diseases (e.g. chronic respiratory diseases), cardiovascular diseases, mastocytosis or clonal mast cell disorders, severe atopic disease, and some concomitant medications (e.g. β-adrenergic blockers and angiotensin-converting enzyme inhibitors).^47^

Before infusion, a medical history should be collected, including details on allergic disorders, atopic status, prior infusion reactions, and concomitant treatments. Based on these assessments, prophylaxis with antihistamines, corticosteroids, or both to reduce the risk of infusion-related reactions can be considered.^46^ Typically, in our experience, prophylaxis should include the use of H1- and H2-receptor antagonists or antihistamines such as ranitidine (150 mg by mouth), diphenhydramine (50 mg by mouth), chlorpheniramine (10 mg by mouth), or pantoprazole (40 mg by mouth) before infusion. Prophylaxis may also include corticosteroids such as dexamethasone (4-8 mg by mouth) or hydrocortisone injections (125 or 250 mg). Newer-class antihistamines, such as loratadine, may also be considered.

Infusion-related reactions to T-DXd, including hypersensitivity and flushing, are common (1%-3% of patients) and were reported in DESTINY-Breast01 in 2.2% of patients (4 of 184), with all cases being low grade (grade 1 or 2) (Table 1).^6^^,^^9^^,^^11^^,^^13^^,^^14^ The rate of T-DXd infusion may be slowed (50% reduction) or temporarily stopped if a patient experiences an infusion-related reaction (grade 1 or grade 2, respectively); the infusion may be permanently stopped if the reaction is severe (grade 3 or 4).^9^, ^10^, ^11^, ^12^, ^13^, ^14^, ^15^^,^^32^ Typical T-DXd-related infusion reactions might include fever and chills, nausea/vomiting, pain, headache, dizziness, dyspnea, or hypotension.^31^

Health care providers should be prepared for a reaction by using the standard protocols at their institutions. Necessary resources to quickly treat an infusion reaction should be readily available. Prompt recognition and treatment are important for reducing the risk of severe symptoms. If anaphylaxis is suspected, local management guidelines should be followed, which might include administration of epinephrine (1 mg/ml) intramuscularly every 5-15 min, i.v. infusion of normal saline (1-2 l at 5-10 ml/kg for the first 5 min), and H1/H2 antagonists.^47^ Infusion-related reactions are a clinically relevant risk for patients receiving T-DXd but can be managed effectively if proper preparations take place.

### Alopecia

Alopecia is a common and usually temporary side-effect of systemic cancer treatment that can be difficult to cope with psychologically and socially.^48^ It is a result of the cytotoxic actions of the chemotherapy agents on rapidly dividing cells in the hair shaft.^48^

In DESTINY-Breast03, alopecia occurred in 36.2% of patients (93 of 257), with most cases [26.5% (68 of 257)] being grade 1 (Table 1).^16^ Limiting the amount of blood flow to the scalp using scalp hypothermia may help block the effects of T-DXd on the hair follicle^49^^,^^50^; however, there are no data available regarding its effectiveness for T-DXd-related alopecia. Scalp cooling causes vasoconstriction and reduces biochemical activity in the hair follicles.^50^ A recent meta-analysis of scalp cooling in randomized clinical trials using chemotherapy found that the relative risk of significant alopecia (i.e. >50% extent of alopecia) was reduced significantly by 46% with scalp cooling relative to no scalp cooling during chemotherapy.^51^ In seven of eight randomized clinical trials, a significant advantage was seen in scalp-cooled patients.^50^ Scalp cooling is mentioned as a consideration for prevention of chemotherapy-induced alopecia for patients with breast cancer.^52^

If a patient chooses this intervention, scalp cooling should be initiated 20-45 min before T-DXd infusion and continued until 20-150 min after the infusion; these timings are based on the guidelines set forth by the European Society for Medical Oncology Clinical Practice Guidelines for chemotherapy-induced alopecia and may need to be modified to optimize effectiveness for T-DXd-related alopecia.^50^ Patients should be educated about the potential side-effects (e.g. headaches, nausea, dizziness) of scalp cooling before receiving this therapy.^49^ Studies on scalp cooling in patients with breast cancer, including patients with metastatic breast cancer who received T-DXd, are ongoing.^53^

Pharmacological interventions to prevent chemotherapy-induced alopecia are currently not approved for use; however, bimatoprost ophthalmic solution and topical minoxidil may be used once treatment has ended to help with regrowth.^50^^,^^54^ Although clinical data suggest that T-DXd-related alopecia occurs in approximately one-third of patients and tends to be grade 1, treatment-induced alopecia can be psychologically and socially distressing for patients; as such, options to prevent or reduce alopecia can be an important consideration in patient care.

### Fatigue

Patients treated with T-DXd may develop fatigue, which can be of grade ≥3 (Table 1).^6^^,^^16^ Cancer-related fatigue may be the result of the cancer or the cancer treatment, and it can be persistent and interfere with usual functioning.^33^^,^^34^

Fatigue is subjective, and patients should be regularly screened for evidence of fatigue.^33^^,^^34^ Patients should be assessed for treatable contributing factors (e.g. pain, depression, insomnia, nutritional deficits, comorbidities, etc.).^33^ A full assessment including clinical history, associated symptoms, fatigue assessments, and existing comorbidities should be conducted.^33^ Screening should be done using validated tools, such as the Numeric Rating Scale for Fatigue.^55^

If a treatable factor is identified, the patient should follow specified treatment guidelines for that factor to help manage and alleviate fatigue.^33^^,^^34^ For other cancer-related fatigue, ongoing patient and caregiver education is necessary to properly manage T-DXd-related fatigue. Patients should be encouraged to stay physically active, if possible, to reduce fatigue-related symptoms; psychosocial interventions (e.g. psychosocial counseling, psychotherapy, and mind–body interventions) should also be considered. Short-term pharmacological intervention could be considered in patients with metastatic cancer.

In our experience, long-term fatigue has not been observed in patients treated with T-DXd. Dose reductions have been effective for improving fatigue, with no cumulative fatigue observed; however, this observation is anecdotal. Managing T-DXd-related fatigue is important for patient well-being since it can hinder usual function, but there are many treatment strategies to help alleviate this AE.

## Management of t-dxd-related aes of interest

### ILD/pneumonitis

A risk of pulmonary AEs, primarily ILD/pneumonitis, has been observed in patients receiving T-DXd and is noted as a warning on the approved labels.^9^, ^10^, ^11^, ^12^, ^13^, ^14^, ^15^ ILD is a large, heterogeneous group of lung disorders that manifests as inflammation and/or fibrosis mainly in the interstitium of the lungs; pneumonitis is a group of disorders characterized by inflammation in the lungs.^32^^,^^56^, ^57^, ^58^ ILD/pneumonitis is a drug-induced toxicity frequently caused by oncologic drugs.^59^

A pooled analysis of eight single-arm, phase I and II, T-DXd monotherapy studies [DS8201-A-J101, DS8201-A-J102 (NCT03366428), DS8201-A-A103 (NCT03368196), DS8201-A-A104 (NCT03383692), DESTINY-Breast01, DESTINY-CRC01 (NCT03384940; DS8201-A-J203), DESTINY-Lung01 (NCT03505710; DS8201-A-U204), and DESTINY-Gastric02 (NCT04014075; DS8201-A-U205)], which included 879 patients with solid tumors (breast, gastric, lung, colorectal, and other tumors) who received 5.4, 6.4, 7.4, or 8.0 mg/kg T-DXd every 3 weeks, found that 15.8% of the total patient population (139 of 879) developed ILD/pneumonitis and that 77.7% of patients (108 of 139) with ILD/pneumonitis experienced grade 1 or 2 events.^60^ Among 245 patients in the pooled analysis who had HER2-positive breast cancer and received 5.4 mg/kg T-DXd every 3 weeks, 15.5% (38 of 245) had drug-related ILD/pneumonitis, with 78.9% of those patients who developed ILD/pneumonitis (30 of 38) having grade 1 or 2 events. In the HER2-positive breast cancer cohort, 2.4% of patients (6 of 245) had fatal events.^60^

In the most recent data for HER2-positive breast cancer from the DESTINY-Breast03 trial, the incidence of any-grade drug-related ILD/pneumonitis with T-DXd was lower at 10.5% (27 of 257) compared with prior DESTINY trials, with no grade 4 or fatal events observed (Table 1).^16^ Multiple factors may have contributed to this overall lower incidence and absence of life-threatening events, including the institution of strict guidelines regarding T-DXd treatment delays and discontinuations and for the treatment of ILD/pneumonitis, and the less heavily treated patient population in the trial.^8^^,^^16^^,^^20^

**Figure 2:**
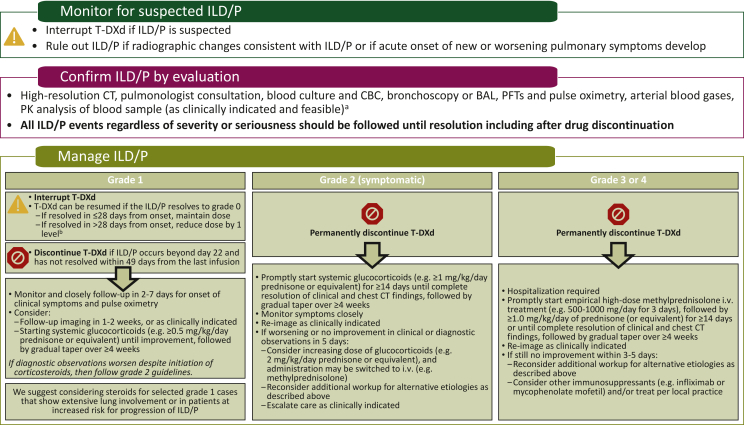
Management of ILD/pneumonitis.^20^^,^^59^^,^^61^^,^^62^ The ‘Manage ILD/P’ portion of this figure is from Figure 3 of Swain SM, Nishino M, Lancaster LH, et al. Multidisciplinary clinical guidance on trastuzumab deruxtecan (T-DXd)-related interstitial lung disease/pneumonitis-Focus on proactive monitoring, diagnosis, and management. *Cancer Treat Rev*. May 2022;106:102378.^62^https://doi.org/10.1016/j.ctrv.2022.102378, Creative Commons license and disclaimer available from https://creativecommons.org/licenses/by-nc-nd/4.0/. BAL, bronchoalveolar lavage; CBC, complete blood count; CT, computed tomography; ILD/P, interstitial lung disease/pneumonitis; i.v., intravenous; PFT, pulmonary function test; PK, pharmacokinetics; q3w, every 3 weeks; T-DXd, trastuzumab deruxtecan. ^a^Evaluations should include high-resolution CT, pulmonologist consultation (infectious disease consultation as clinically indicated), blood culture and CBC (other blood tests should be considered as needed), bronchoscopy and BAL if clinically indicated and feasible, PFTs and pulse oximetry, arterial blood gases if clinically indicated, and one blood sample for PK analysis as soon as ILD/P is suspected if feasible. ^b^See Table 3 for the dose reduction levels.

It is of the utmost importance to monitor and assess patients for ILD/pneumonitis to reduce the risk of fatal outcomes (Figure 2).^59^^,^^61^ In 2019, guidelines for the management and monitoring of T-DXd-related ILD/pneumonitis were published and incorporated into clinical trials of T-DXd^6^; these were updated in 2021.^20^ These guidelines stress the importance of proactive management and monitoring to help identify and treat ILD/pneumonitis as effectively as possible; the guidelines provide more details around steroid dosing, duration, and timing of taper for better management (Figure 2). Patients should be educated on the signs and symptoms of ILD/pneumonitis and instructed to report changes in or onset of these symptoms to their health care team immediately. These signs or symptoms include cough, shortness of breath, fever, or any other new or worsening respiratory symptoms. Regular monitoring is also warranted while a patient is being treated with T-DXd, including computed tomography (CT) scans (CT or high-resolution CT). CT scans should be carried out before initiation of T-DXd treatment and at least every 9-12 weeks during treatment (i.e. consistent with the typical schedule for monitoring therapeutic response).^62^ For patients who experience ILD/pneumonitis, follow-up CT imaging is recommended every 1-2 weeks or as clinically indicated.^6^^,^^20^^,^^62^ Consultation with a pulmonary specialist for monitoring and treatment recommendations is necessary if T-DXd-induced ILD/pneumonitis is suspected or develops.

**Table 3:** Dosing of T-DXd and dose levels for dose reductions^9^, ^10^, ^11^, ^12^, ^13^, ^14^, ^15^

Dose	Breast cancer indication
Starting dose	5.4 mg/kg q3w
First-level reduction	4.4 mg/kg q3w
Second-level reduction	3.2 mg/kg q3w
Further reduction	Discontinue

q3w, every 3 weeks; T-DXd, trastuzumab deruxtecan.

Current T-DXd ILD/pneumonitis guidelines recommend starting steroids promptly upon detection for grade ≥2 ILD/pneumonitis and suggest consideration of steroid treatment of grade 1 cases (Figure 2).^6^^,^^20^^,^^59^^,^^62^ Steroid treatment may be warranted for some patients with grade 1 ILD/pneumonitis, including those with extensive lung involvement or those who are at increased risk for progression of ILD/pneumonitis.^62^ Treatment of ILD/pneumonitis with systemic steroids may help mitigate progression to higher-grade ILD/pneumonitis.^62^

Rechallenging patients with asymptomatic ILD/pneumonitis that has not completely resolved is an interesting approach to consider in the future. Currently, rechallenge is only recommended for patients with grade 1 ILD/pneumonitis that resolves; patients with grade ≥2 ILD/pneumonitis should permanently discontinue T-DXd.^20^ Further studies will be needed to evaluate rechallenge in asymptomatic patients maintaining grade 1 ILD/pneumonitis after drug hold; however, in our experience, if resolution of the grade 1 ILD/pneumonitis is achieved, treatment with T-DXd may be resumed. Cumulative toxicity of T-DXd has not been observed in clinical trials; data from the pooled analysis suggested that the risk for adjudicated drug-related ILD/pneumonitis exists primarily in the first year of therapy. The median time to ILD/pneumonitis onset was 5.5 months (range, <0.1-46.8 months) among patients with all tumor types and doses and 5.6 months (range, 1.1-20.8 months) among patients with HER2-positive metastatic breast cancer receiving 5.4 mg/kg every 3 weeks.^60^^,^^63^ ILD/pneumonitis remains a significant risk for patients treated with T-DXd; however, as proactive management and treatment improves, the incidence of these events may lessen.

### Left ventricular dysfunction

The risk of decreased left ventricular ejection fraction (LVEF) has been previously reported with T-DXd treatment; although the occurrences in T-DXd clinical trials were generally asymptomatic,^6^^,^^41^ it is a stated warning on approved labels.^9^, ^10^, ^11^, ^12^, ^13^, ^14^, ^15^ In the DESTINY-Breast03 trial, decrease in ejection fraction or left ventricular dysfunction occurred in 2.7% of patients (7 of 257) treated with T-DXd; all cases were low grade (grade 1 or 2) and asymptomatic.^16^ Because of this risk, LVEF should be assessed before beginning treatment with T-DXd and at regular intervals during treatment (e.g. every 3-4 months) if asymptomatic. In the case of LVEF decrease, T-DXd should be held or discontinued per the direction on the labels depending on the severity of the LVEF decrease.^9^, ^10^, ^11^, ^12^, ^13^, ^14^, ^15^ T-DXd should be permanently discontinued if congestive heart failure occurs. The rates of decreased LVEF, along with other cardiac toxicities, associated with T-DXd are low, but clinicians should assess patients regularly during T-DXd treatment and consult with a cardiologist if warranted.

## Future directions

Controlling AEs occurring in patients with breast cancer treated with T-DXd can allow patients to experience maximum treatment benefit. To that end, there are several areas in which additional data and further research are needed. An important question to be answered in future studies for optimizing T-DXd use and AE management is the possible validity of rechallenging patients with asymptomatic ILD/pneumonitis that has not resolved, or patients with grade 2 ILD/pneumonitis that has fully resolved. Ideally, these studies would seek to determine the optimal frequency of imaging assessment to detect asymptomatic ILD/pneumonitis in patients without disease in the lung (e.g. early-stage breast cancer), the best techniques (e.g. low-dose high-resolution CT scan), and the risk factors for more severe toxicity.

Additionally, optimization of prophylaxis and management of T-DXd-related nausea and vomiting could be investigated in prospective or retrospective studies. Whether short-acting (e.g. ondansetron) and long-acting (e.g. palonosetron) 5-HT_3_ receptor antagonists are more appropriate as prophylaxis should also be investigated to confirm efficacy and preferences. More precise studies into the optimal intervention for delayed-onset nausea and vomiting should also be conducted for the same reason. Many of these questions could be answered using real-world data, which could also confirm whether the toxicity profile of T-DXd in real-world practice is similar to that observed in clinical trials.

Finally, several trials in patients with breast cancer are ongoing to investigate T-DXd in combination with other agents, including durvalumab, paclitaxel, tucatinib, pertuzumab, anastrozole, nivolumab, pembrolizumab, and ceralasertib.^64^, ^65^, ^66^, ^67^, ^68^, ^69^, ^70^ Understanding both the efficacy and safety AE profile of T-DXd when used in combination with other anticancer agents will be important. In an interim analysis of the phase Ib trial of T-DXd with nivolumab in patients with advanced/metastatic HER2-positive or HER2-low-expressing breast cancer (NCT03523572; DS8201-A-U105; *N* = 48), the types and rates of reported AEs were consistent with those seen with T-DXd monotherapy.^71^ The most common AEs were nausea (54.2%), fatigue (45.8%), and alopecia (41.7%); ILD/pneumonitis was reported in 10.4% (5 of 48; 1 fatal case); however, these findings are limited by the relatively short duration of T-DXd treatment (median: HER2-positive, 6.5 months; HER2-low, 6.3 months) and follow-up (median: HER2-positive, 7.0 months; HER2-low, 6.9 months).^71^ These early results are encouraging in that it may be possible to combine T-DXd with other therapies to further enhance outcomes without incurring additional toxicity.

### Conclusions

T-DXd is a relatively recent treatment option, and information can be gained from understanding the usage, monitoring, and management practices of other health care providers and institutions with experience using T-DXd. The robust efficacy data related to T-DXd and the expectation of longer treatment duration will require more stringent symptom control to avoid negative impacts on quality of life. While the majority of T-DXd-related AEs are familiar to oncologists, management of ILD/pneumonitis and prevention of severe ILD/pneumonitis in particular warrants further education. Most T-DXd-related AEs can be managed safely and effectively by multidisciplinary teams. It is our hope that by increasing familiarity with the management, diagnosis, and monitoring of T-DXd-associated AEs, more patients will be able to maximize T-DXd treatment benefit.
